# Effect of cancer on outcome of COVID-19 patients: a systematic review and meta-analysis of studies of unvaccinated patients

**DOI:** 10.7554/eLife.74634

**Published:** 2022-02-16

**Authors:** Giulia Di Felice, Giovanni Visci, Federica Teglia, Marco Angelini, Paolo Boffetta

**Affiliations:** 1 Department of Medical and Surgical Sciences, University of Bologna Bologna Italy; 2 IRCCS Azienda Ospedaliero-Universitaria di Bologna Bologna Italy; 3 Stony Brook Cancer Center, Stony Brook University Stony Brook United States; Roswell Park Comprehensive Cancer Center United States; McGill University Canada

**Keywords:** SARS-CoV-2, COVID-19, cancer, mortality, disease severity, meta-analysis, Human

## Abstract

**Background:**

Since the beginning of the SARS-CoV-2 pandemic, cancer patients affected by COVID-19 have been reported to experience poor prognosis; however, a detailed quantification of the effect of cancer on outcome of unvaccinated COVID-19 patients has not been performed.

**Methods:**

To carry out a systematic review of the studies comparing the outcome of unvaccinated COVID-19 patients with and without cancer, a search string was devised which was used to identify relevant publications in PubMed up to December 31, 2020. We selected three outcomes: mortality, access to ICU, and COVID-19 severity or hospitalization. We considered results for all cancers combined as well as for specific cancers. We conducted random-effects meta-analyses of the results, overall and after stratification by region. We also performed sensitivity analyses according to quality score and assessed publication bias.

**Results:**

For all cancer combined, the pooled odds ratio (OR) for mortality was 2.32 (95% confidence interval [CI] 1.82–2.94, I^2^ for heterogeneity 90.1%, 24 studies), that for ICU admission was 2.39 (95% CI 1.90–3.02, I^2^ 0.0%, 5 studies), that for disease severity or hospitalization was 2.08 (95% CI 1.60–2.72, I^2^ 92.1%, 15 studies). The pooled mortality OR for hematologic neoplasms was 2.14 (95% CI 1.87–2.44, I^2^ 20.8%, 8 studies). Data were insufficient to perform a meta-analysis for other cancers. In the mortality meta-analysis for all cancers, the pooled OR was higher for studies conducted in Asia than studies conducted in Europe or North America. There was no evidence of publication bias.

**Conclusions:**

Our meta-analysis indicates a twofold increased risk of adverse outcomes (mortality, ICU admission, and severity of COVID-19) in unvaccinated COVID-19 patients with cancer compared to COVID-19 patients without cancer. These results should be compared with studies conducted in vaccinated patients; nonetheless, they argue for special effort to prevent SARS-CoV-2 infection in patients with cancer.

**Funding:**

No external funding was obtained.

## Introduction

Since the emergence of SARS-CoV-2, many studies have been conducted on the outcomes of COVID-19, in order to identify factors associated with a higher death rate and a more severe infection course. Some groups of patients at increased risk of severe COVID-19, morbidity, and mortality have been identified, including elderly patients, and those with comorbidities, such as hypertension, diabetes, chronic kidney disease, or COPD (Fang et al., 2020). Cancer patients are also a high-risk group due to their compromised immune systems and vulnerability to infection resulting from their disease and the treatments (Kamboj and Sepkowitz, 2009).

It is generally assumed that cancer patients are at higher risk for severe COVID-19 and death attributed to COVID-19 (Rüthrich et al., 2021). However, cancer encompasses a very heterogeneous group of diseases with a diverse range of subtypes and stages. In addition, not all cancers are equal in terms of incidence, prognosis, and treatment. This must be taken into account when the type of cancer is not specified (Lee et al., 2020). For this reason, although descriptions and analyses of risk factors, clinical courses, and mortality in cancer patients infected with SARS-CoV-2 have been reported, a quantitative assessment of the effect of cancer in patients with COVID-19 would be important to guide clinical decision-making.

We aimed at conducting a systematic review of the epidemiological features of the studies of COVID-19 in cancer patients conducted before the implementation of vaccination campaigns, and to provide a quantitative estimate of the risk of severe infection course and mortality in COVID-19 patients with cancer compared to COVID-19 patients without cancer. We decided to restrict our review to studies of unvaccinated patients because (i) they provide the clearest picture of the effect of cancer on outcome of COVID-19 patients, and (ii) the full effect of vaccination might not have been yet captured by available studies.

## Materials and methods

This systematic review was conducted according to the PRISMA statement (Moher et al., 2009). We submitted the protocol (available as Supplementary file 1) to the PROSPERO Registry. To carry out the systematic review of the scientific literature, the following string was used for the PubMed database:

(neoplas*[TIAB] OR tumor*[TIAB] OR cancer*[TIAB] OR malignancy [TIAB]) AND (2019 novel coronavirus[TIAB] OR COVID-19[TIAB] OR COVID19[TIAB] OR SARS-CoV-2[TIAB] OR 2019-nCoV[TIAB]).

In order to restrict the review to study populations on unvaccinated COVID-19 patients, we included papers published in peer-reviewed journals up to December 31, 2020. We excluded abstracts and non-peer-reviewed reports, articles in languages other than English, and studies including children. We also excluded reviews, meta-analysis and case reports, and studies with less than 50 patients or less than 10 events. Finally, we excluded studies in which diagnosis of SARS-CoV-2 infection was not made by PCR testing.

The articles were independently reviewed and abstracted by two pairs of reviewers (GDF and MA; GV and FT), on the basis of title, abstract, and full text; the disagreement between the authors of the reviews (6.1% of all studies) and was resolved through discussion with a fifth reviewer [PB].

We selected the following outcomes: mortality, ICU admission, severity of COVID-19 symptoms, and hospitalization: we combined these latter two outcomes because the definition of severity was heterogeneous across studies and the number of available studies was low. We excluded from the review studies addressing the impact of SARS-CoV-2 infection on prevention, diagnosis, and treatment of cancer patients, for example, studies comparing cancer patients with and without SARS-CoV-2 infection, as well as studies on the oncogenic effect of the virus, for example, analyses of cancer-related alterations. In addition, we carried out a back-search by inspecting the lists of references of articles selected for the review.

**Figure 1.**
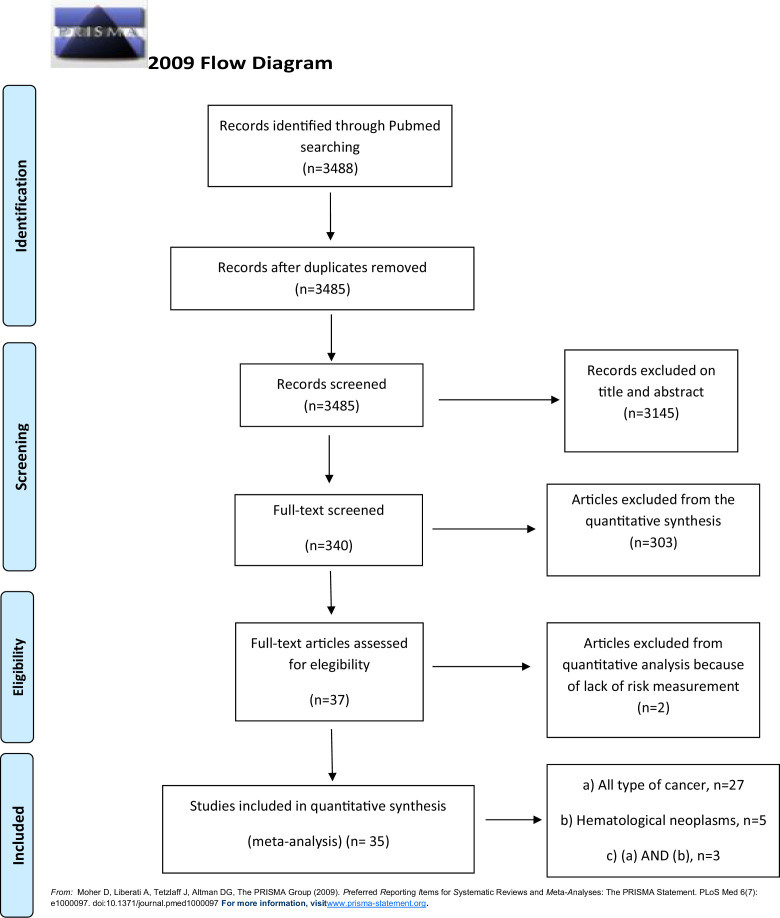
Flowchart for the identification of articles for the meta-analyses (PRISMA).

Figure 1 shows the flowchart for selection of the studies. Details on the studies retained in each step of the process are available from the authors.

We abstracted the following parameters from the articles retained for the review: country, sample size, number of persons affected by cancer and by SARS-CoV-2 infection, cancer type and comparison group (patients without cancer or patients with a different type of cancer), outcome, and risk estimate (relative risk or odds ratio [OR]) with 95% confidence interval (CI). If the risk estimate or the CI were not reported in the publication, we calculated them from the raw data, if possible. We also performed a quality assessment (QA) based on a modified version of CASP score (Critical Appraisal Skills Programme, 2018), that included 10 criteria (Supplementary file 2).

### Statistical analysis

We conducted random-effects (DerSimonian and Laird, 1986) meta-analyses of the risk estimates for the combinations of cancers and outcomes with more than five independent results. We also conducted stratified meta-analyses according to geographic region, to explore potential sources of heterogeneity, that we quantified using the I^2^ test (Higgins and Thompson, 2002).

To evaluated results stability, we performed sensitivity analyses by quality score and repeated the meta-analysis after excluding one study at a time. We also conducted secondary analyses excluding studies with results calculated on the basis of raw data. Furthermore, we considered the funnel plot and performed the Egger’s regression asymmetry test to assess publication bias (Egger et al., 1997).

Finally, we conducted a cumulative meta-analysis, based on date of publication of subsequent studies.

Analyses were performed by STATA16 program (StataCorp, 2019), using specific commands metan, metabias, and metafunnel.

## Results

We identified a total of 3488 publications from the literature search and excluded 3 because they were duplicates. We screened the titles and abstracts of 3485 articles: we excluded 3145 of them because not relevant (Figure 1), and retained 340 articles as potentially eligible.

After reviewing the full-texts, we excluded 303 articles because these did not meet the inclusion criteria, and included the remaining 37 studies in the review: we finally included 35 of them in the quantitative synthesis.

Among the 35 studies, 30 reported results for all cancers combined, and 8 for hematologic neoplasms (3 of these reported both sets of results). Results for other specific cancers were sparse, and we could not conduct meta-analyses of them. Out of the 35 studies, 13 were from Europe, 11 from North America (all from the United States), and 11 from Asia (9 from China and 2 from Iran). Fifteen studies were considered good quality (CASP score>9.5), 19 studies were of moderate quality (9.5≥CASP score>6), whereas 1 was considered inadequate (CASP score≤6).

Tables 1 and 2 show the details of the studies included in the analysis.

**Table 1.** Selected characteristics of studies included in the meta-analysis – all cancers.

References	Country	N patients	N outcomes	Quality score	Comparison	Outcome	RR/OR*	95% CI
Dai et al., 2020	China	641	105	9.25	Internal	Mortality	2.34	1.15–4.77
						ICU	2.84	1.59–5.08
						Severity	2.79	1.75–4.44
Haase et al., 2021	Denmark	323	15	11.5	Internal	Mortality	3.18	1.66–6.09
Meng et al., 2020	China	436	109	9.5	Internal	Mortality	2.98†	1.76–5.05
Sun et al., 2020	USA	323	67	10.5	Internal	Mortality	5.67†	1.49–21.58
						ICU	1.91†	0.90–4.06
						Hospitalization	2.16†	1.12–4.17
Nogueira et al., 2020	Portugal	20,293	611	9.25	Internal	Mortality	1.48	1.07–2.05
Zandkarimi et al., 2020	Iran	1831	32	6.5	Internal	Mortality	3.57	1.82–7.02
Zhao et al., 2020	China	539	23	6.5	Internal	Mortality	*3.23*	*1.39–7.51*
Harrison et al., 2020	USA	31,461	1966	9.75	Internal	Mortality	0.87†	0.72–1.09
Gupta et al., 2020	USA	2215	112	7.5	Internal	Mortality	*2.20*	*1.50–3.22*
Ganatra et al., 2020	USA	2476	195	9.25	Internal	Mortality	*3.53*	*2.95–4.23*
						Severity	*3.75*	*3.17–4.44*
						Hospitalization	*2.78*	*2.37–3.26*
Westblade et al., 2020	USA	2294	100	8.5	Internal	Mortality	*1.29*	*1.04–1.61*
						Severity	*0.76*	*0.57–1.01*
Wang et al., 2020	USA	16,570	1200	11	Internal	Mortality	*3.20*	*2.89–3.55*
						Hospitalization	*2.85*	*2.63–3.09*
Cherri et al., 2020	Italy	2039	53	11	Internal	Mortality	2.22†	1.25–3.94
Görgülü and Duyan, 2020	Turkey	483	75	11	Internal	Mortality	1.81†	0.88–3.72
						ICU	*2.14*	*1.26–3.63*
Jiménez et al., 2020	Spain	1549	103	9.75	Internal	Mortality	4.29†	2.40–7.67
Thompson et al., 2020	UK	470	87	10	Internal	Mortality	2.20†	1.27–3.81
Li et al., 2020	China	1859	65	9	External	Mortality	1.59†	0.94–2.68
Shoumariyeh et al., 2020	Germany	78	39	8.5	External	Mortality	*1.01*	*0.41–2.49*
						Severity	*1.15*	*0.61–2.17*
Mehta et al., 2020	USA	1308	218	8.5	External	Mortality	*2.38*	*1.69–3.35*
Rogado et al., 2020	Spain	42,495	45	8	External	Mortality	*4.82*	*2.67–8.71*
Brar et al., 2020	USA	585	117	9.5	External	Mortality	0.98	0.58–1.66
						Severity	0.80	0.57–1.13
Zhang et al., 2020b	China	217	112	9.5	External	Mortality	*4.83*	*2.87–8.12*
						ICU	*2.60*	*1.87–3.62*
						Severity	*1.43*	*1.09–1.87*
Sorouri et al., 2020	Iran	159	53	10	External	Mortality	3.27†	0.93–11.55
						ICU	1.52†	0.56–4.12
Lunski et al., 2021	USA	5145	312	9.5	External	Mortality	2.03†	1.44–2.87
Atalla et al., 2021	USA	339	27	10.5	Internal	Hospitalization	*3.34*	*1.81–6.16*
Cheng et al., 2020	China	1476	29	6	Internal	Severity	*2.14*	*0.97–4.73*
Song et al., 2021	China	961	21	7	Internal	Severity	*2.77*	*1.16–6.62*
Liang et al., 2020	China	1590	18	8.75	Internal	Severity	4.07†	1.23–13.45
Bauer et al., 2021	USA	1449	108	8.5	Internal	Severity	1.72†	1.11–2.67
Tian et al., 2020	China	751	232	9	External	Severity	*3.75*	*2.71–5.19*

* Results derived from data reported in the publication are in italics.

† Risk estimates derived from multivariate analysis.

RR: Relative Risk; OR: Odds Ratio; CI: Confidence interval; ICU: Intensive Care Unit.

**Table 2.** Selected characteristics of studies included in the meta-analysis – *hematological tumors.*

Author – Year	Country	Sample size (n)	Cancer (n)O number of cancer (%)	Quality assessment (CASP)	Comparison	Outcome	RR/OR*	IC
Dai et al., 2020	China	641	9	9.25	Internal	Mortality	9.07	2.16–38.13
Haase et al., 2021	Denmark	323	13	11.5	Internal	Mortality	1.83	0.85–3.93
Meng et al., 2020	China	327	16	9.5	Internal	Mortality	2.83†	0.96–8.32
Yigenoglu et al., 2021	Turkey	1480	740	10.5	External	Mortality	*2.20*	*1.93–2.50*
Shah et al., 2020	UK	1183	68	9.75	External	Mortality	1.74†	1.12–2.71
Sanchez-Pina et al., 2020	Spain	92	39	10.25	External	Mortality	6.65†	1.87–23.67
Passamonti et al., 2020	Italy		536	11	External	Mortality	2.04†	1.77–2.35
Cattaneo et al., 2020	Italy	204	102	9	External	Mortality	*2.10*	*1.14–3.85*

* italics characters when calculated manually.

† Risk estimates derived from multivariate analysis.

CASP: Critical Appraisal Skills Programme; RR: Relative Risk; OR: Odds Ratio; IC: Interval Confidence; ICU: Intensive Care Unit.

**Figure 2.**
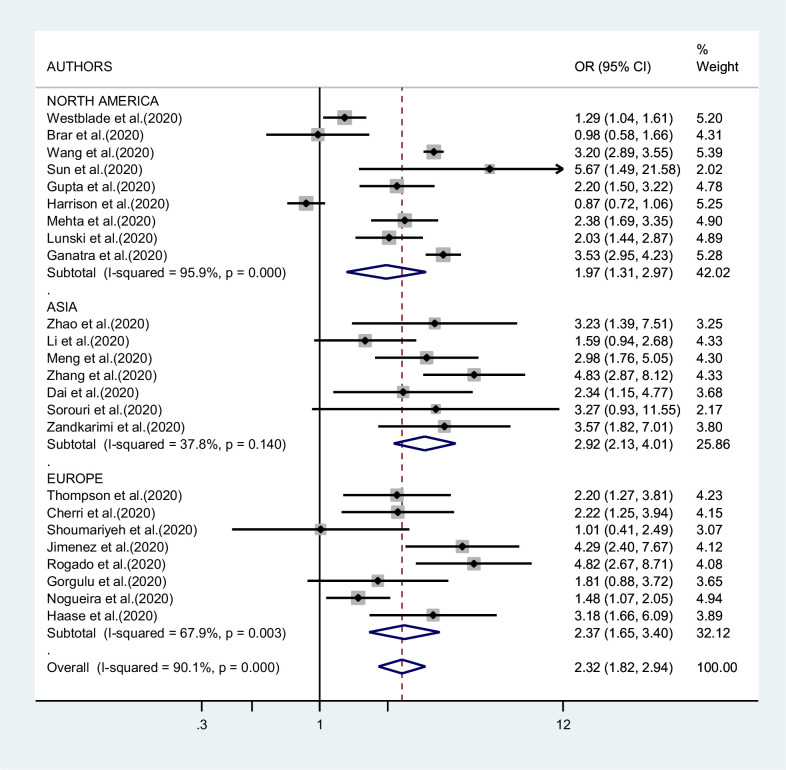
Forest plot – all types of cancer – outcome 1.

**Figure 3.**
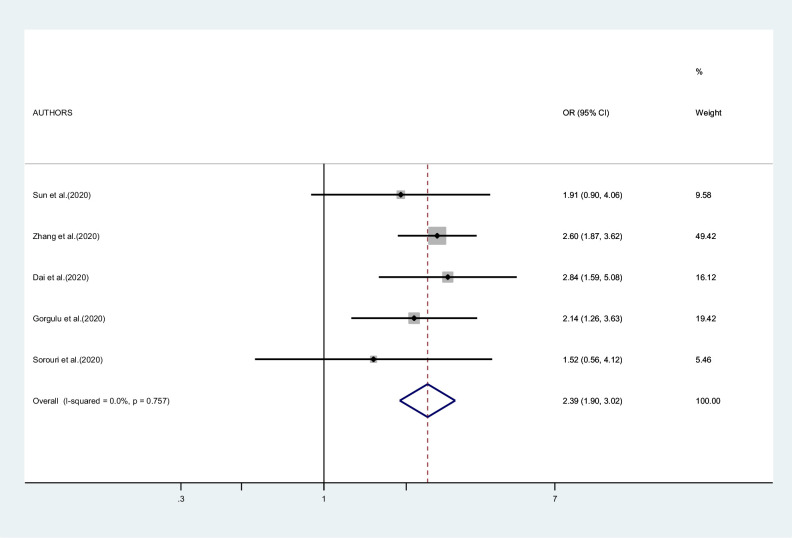
Forest plot – all types of cancer – outcome 2.

**Figure 4.**
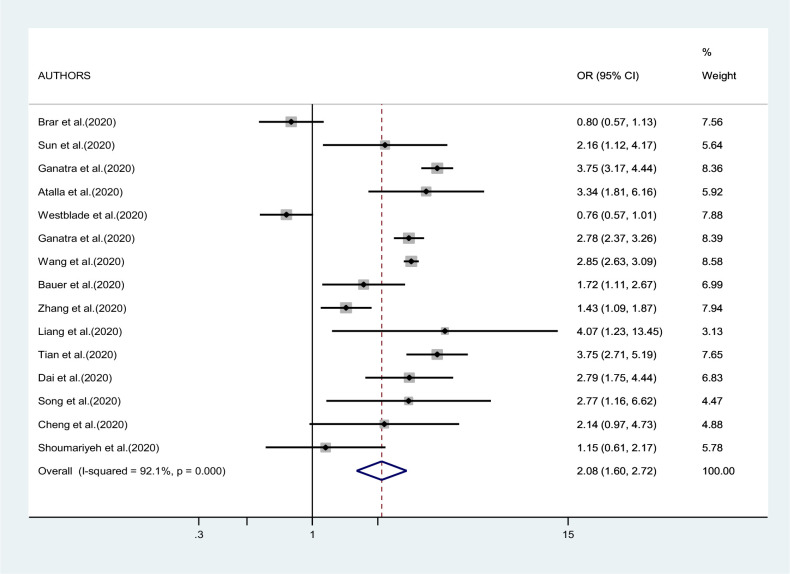
Forest plot – all types of cancer – outcome 3 or 4.

Figures 2—4 report the results of the meta-analyses of studies of COVID-19 patients with all cancers combined compared to patients without cancer, for mortality, admission to ICU, and hospitalization or severity of COVID-19, respectively. The pooled OR for mortality was 2.32 (95% CI 1.82–2.94, I^2^ 90.1%), that for ICU admission was 2.39 (95% CI 1.90–3.02, I^2^ 0.0%), and that for hospitalization/severity of disease was 2.08 (95% CI 1.60–2.72, I^2^ 92.1%).

In the analysis by geographic region (Figure 2), the association between SARS-CoV-2 infection and mortality in cancer patients was stronger, and less heterogeneous, in studies from Asia (OR 2.92; 95% CI 2.13–4.01, I^2^ 37.8%) than in studies from either Europe (OR 2.37; 95% CI 1.65–3.40; I^2^ 67.9%) or North America (OR 1.97; 95% CI 1.31–2.97; I^2^ 95.9%, respectively). Too few studies were available on the other outcomes to justify a meta-analysis stratified by region of origin.

The cumulative meta-analysis, based on date of publication of subsequent studies of mortality (all types of cancer), showed a stronger association in the studies published before July 2020 than in studies published later (results not shown in detail).

**Figure 5.**
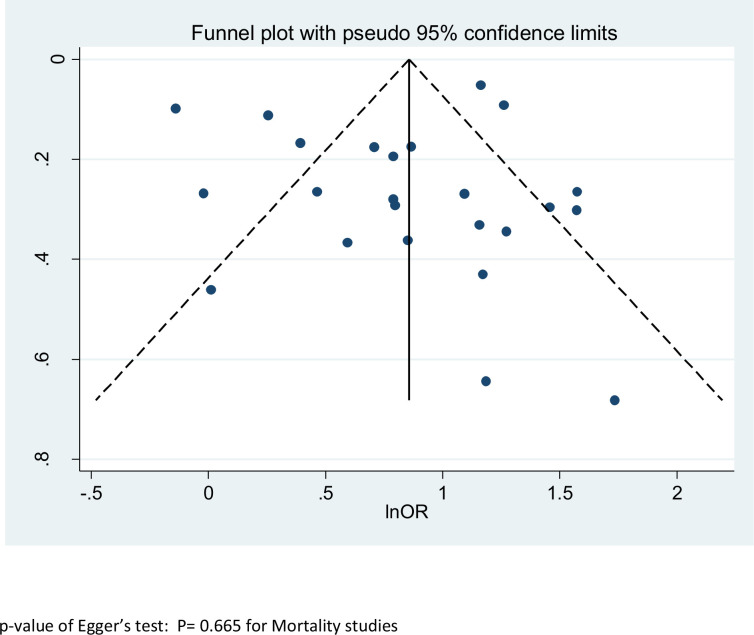
Funnel plot of Egger’s test to assess publication bias – all types of cancer – outcome 1.

As shown in Figure 5, we found no evidence of publication bias in the meta-analysis concerning mortality (p value of Egger’s test 0.67). The number of studies included in the other meta-analyses was too low to yield meaningful results on publication bias.

**Figure 6.**
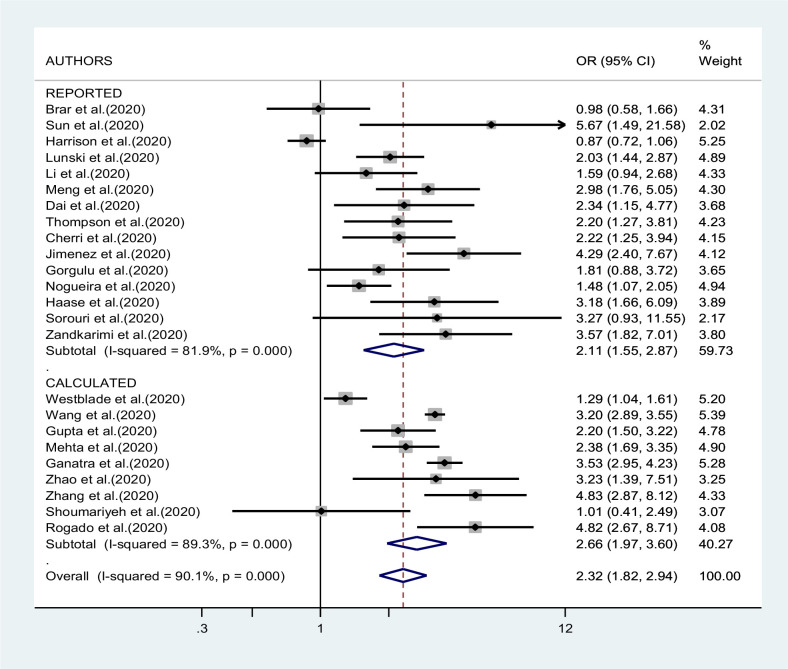
Forest plot – all types of cancer – outcome 1 – reported versus calculated OR.

In the sensitivity analysis based on QA, the pooled OR of mortality results of studies with acceptable quality was not different from that of results of good-quality studies: OR 2.25 (95% CI 1.73–2.94) versus OR 2.50 (95% CI 1.47–4.26). When we repeated the analysis after excluding one study at a time, we did not identify a major effect of any single study; in particular, the exclusion of the only study that suggested a negative association between cancer and mortality (Harrison et al., 2020) yielded a pooled OR of 2.41 (95% CI 1.95–2,99, I^2^ 85.5%). The association with mortality was less pronounced in studies whose results were reported by the authors (OR 2.11; 95% CI 1.55–2.87) compared to studies whose results were calculated by us (OR 2.66; 95% CI 1.97–3.60), although the difference was not statistically significant (Figure 6).

**Figure 7.**
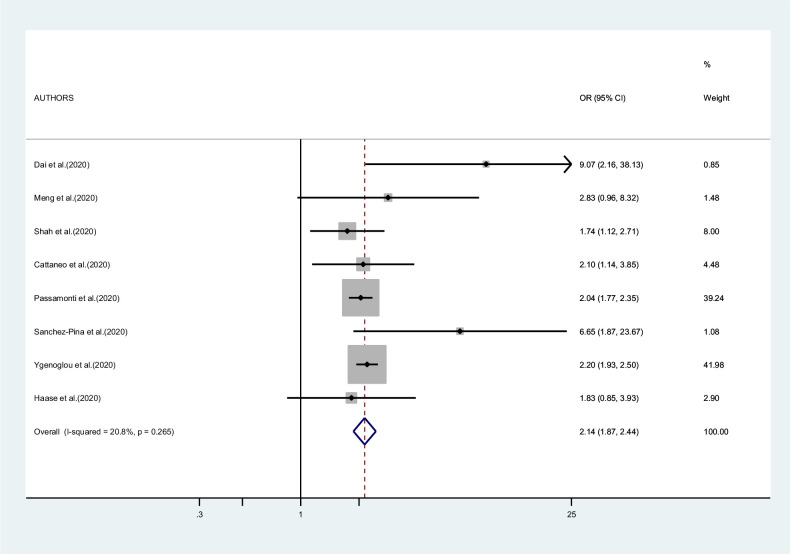
Forest plot – hematological neoplasms – outcome 1.

Figure 7 presents the results of the meta-analysis of eight studies on mortality in patients with hematologic neoplasms. The pooled RR was 2.14 (95% CI 1.87–2.44, I^2^ 20.8%). Results for other outcomes (admission to ICU, hospitalization, and severity of symptoms) were too sparse to conduct a meta-analysis.

**Figure 7—figure supplement 1.**
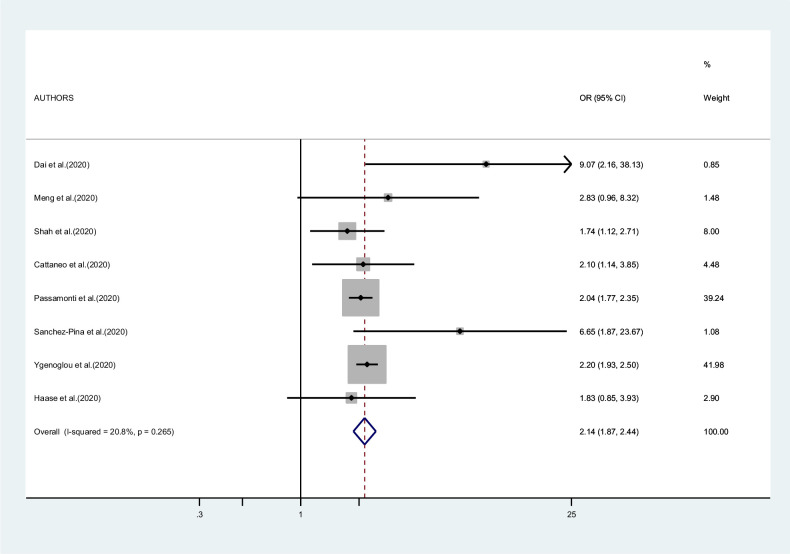
Forest plot – hematological neoplasms – outcome 2, 3, or 4.

## Discussion

Since the beginning of the SARS-CoV-2 pandemic, cancer patients affected by COVID-19 have been identified to be at increased risk of poor prognosis, together with other vulnerable categories of patients as those affected by cardiovascular disease, diabetes, kidney injury, obesity, or stroke (Hu et al., 2020).

However, how much SARS-CoV-2 infection resulted in more severe outcomes in cancer patients compared to patients without cancer and what caused their worse clinical course has not been fully clarified. In a previous editorial, some of us addressed the issue of the different interactions that COVID-19 and cancer may have (Hainaut and Boffetta, 2021). On one hand, it is interesting to study how COVID-19 evolves in patients with cancer, by assessing whether the infection in these patients has a more severe course than in a control group affected by the infection but without cancer. On the other hand, it is important to identify the effects that the pandemic itself has determined in patients with cancer, including reduced access to treatment, delay in diagnosis for postponed screening, increased time between follow-up visits, and change in treatment organization. Acquiring more severe infection could be due to both components.

In this systematic review and meta-analysis, we focused on the effect that cancer had in patients with COVID-19 compared with those without cancer in terms of mortality, ICU access, and severity of COVID-19 (hospitalization or severity of symptoms). We found that patients with cancer and SARS-CoV-2 infection have a twofold higher risk of experiencing these adverse outcomes compared to non-cancer patients. Our results are in agreement with those of the meta-analysis by Venkatesulu et al., 2020, who included a smaller number of studies, mainly from China, and reported an OR of 2.54 (95% CI 1.47–4.42) for mortality in cancer patients with concurrent COVID-19, compared to non-cancer patients. Similar to our results, these authors also reported a stronger association in studies from China than in those from other regions. Our results are also similar to those by Zhang et al., 2020a, who reported a meta-analysis of five studies from China, yielding a meta-OR of 2.63, with limited heterogeneity. Compared to these early reports, we included more studies, which should lead to a more robust and precise risk estimate.

The higher risk of mortality in studies from China compared to those from other countries could be explained by the fact that some of the studies from China were conducted during the very early phase of the infection, when diagnosis and treatment for SARS-CoV-2 might have been delayed, resulting in higher death rate. This interpretation is reinforced by the results of the cumulative meta-analysis that showed a stronger effect detected in the early studies compared to later studies.

Our summary results on the risk of ICU admission and severity of COVID-19 indicated a somehow weaker association than that reported by other authors. An early meta-analysis reported a threefold increase for ICU admission, an almost fourfold increase for a SARS-CoV-2 infection classified as severe, and a fivefold increase in being intubated (ElGohary et al., 2020). The fact that our values are lower might be explained by the inclusion of studies conducted when management of cancer patients with SARS-CoV-2 infection was more effective.

Immunosuppression and impaired T-cell response due to therapies may underlie the worse outcome in hematologic cancer diseases, even if some authors suggested that the attenuated inflammatory response in hematological patients can protect from severe COVID-19 morbidity (Vijenthira et al., 2020). The results of our meta-analysis confirm a higher mortality from COVID in patients with hematological neoplasms compared to non-neoplastic patients, with limited heterogeneity, with a pooled risk estimate similar to that for all cancers combined.

We were not able to derive pooled results for other specific cancers. Results for patients with hematologic and solid neoplasms were compared in some individual studies. In particular, Desai et al., 2021 reported a higher mortality in the former group, but the comparison was not adjusted for age and type of therapy.

Although our study provides the most precise measure to date of the effect of cancer in COVID-19 patients, it suffers from some limitations. Many studies included in our analysis did not provide results adjusted for important determinants such as sex, age, comorbidities, and therapy. As mentioned above, we were not able to analyze specific cancers other than hematologic neoplasms, because results were too sparse.

In conclusion, our meta-analysis confirms, by giving a more precise and accurate estimation, evidence to the hypothesis of an association in COVID-19 patients between cancer (and more specific hematologic neoplasm) and a worst outcome on mortality, ICU admission, and severity of COVID-19.

Future studies will be able to better analyze this association for different subtypes of cancer, and to evaluate whether the effects identified before vaccination are attenuated vaccinated patients.

## Data Availability

All data generated or analysed during this study are included in the manuscript and supporting file. Dataset has been deposited on Dryad (https://doi.org/10.5061/dryad.00000004q). The following dataset was generated: VisciG Di FeliceG TegliaF AngeliniM BoffettaP 2021Data from: Effect of SARS-CoV-2 infection on outcome of cancer patients: A systematic review and meta-analysis of studies of unvaccinated patientsDryad Digital Repository10.5061/dryad.00000004qPMC895628435171096

## Additional files

Supplementary file 1. Protocol.

Supplementary file 2. Quality Assessment.

Reporting standard 1. PRISMA checklist.
