# Supplementary material for: Effect of cancer on outcome of COVID-19 patients: a systematic review and meta-analysis of studies of unvaccinated patients
Source: eLife. 2022 Feb 16;11:e74634. doi: 10.7554/eLife.74634 (PMC8956284; doi:10.7554/eLife.74634)
Supplement: Supplementary file 1. [file elife-74634-supp1.docx]

Supplementary File 1. Protocol (submitted to PROSPERO registry)

**SarsCov2 infection on unvaccinated cancer patients: evaluation of disease severity– A Systematic Review and Meta-Analysis**

**Study Protocol**

**9 August 2021**

***Paolo Boffetta University of Bologna, Bologna, Italy***

***Stony Brook University New York, NY, USA***

paolo.boffetta@unibo.it

***Giulia Di Felice University of Bologna, Bologna, Italy***

giulia.difelice@studio.unibo.it

Corresponding author and guarantor:

Paolo Boffetta, MD, MPH

Department of Medical and Surgical Sciences (DIMEC)
Bologna University Alma Mater Studiorum
Via Massarenti 9, 40138 Bologna

**Sources of financial support**

Internal resources of participating institutions

**Rationale**

SarsCov2 infection can occur asymptomatically, paucisymptomatically, or severely.

Age and comorbidities affect the course of the disease.

Patients with cancer have been identified as frail patients and appeared to be highly susceptible to develop severe forms of COVID-19.

**Objective**

To conduct a systematic review and meta-analysis of epidemiological studies comparing the severity of COVID-19 infection in unvaccinated subjects with cancer versus unvaccinated subjects without cancer.

**Eligibility criteria**

*Study design*

- Case-control-studies and cohort-studies (no case reports, no reviews and meta-analyses)

*Participants*

- Unvaccinated adults with SarsCov2 infection, both sexes, all countries

*Exposure*

- Malignant disease in adults

*Comparison*

- Unvaccinated subjects without cancer or with a different type of cancer

*Outcome*

- Severity of SarsCov2 infection (death, Intensive Care Unit admission, Severity of symptoms or Mechanical controlled ventilation, hospitalization)

*Publication type*

- Papers published or accepted for publication in peer-reviewed journals (no abstracts or unpublished reports)

*Language and time*

- English language
- From 1/1/2019 to 12/31/2020

**Information sources**

Electronic databases of medical literature:

- MEDLINE (PubMed)

**Search strategy**

The following keywords will be used:

1. **Neoplas***
2. **Tumor***
3. **Cancer***
4. **Malignancy**
5. **2019 novel coronavirus**
6. **COVID-19**
7. **COVID19**
8. **SARS-CoV-2**
9. **2019-nCoV**

with the following logical relations: (“1” OR “2” OR “3” OR “4”) AND (“5” OR “6” OR “7” OR “8” OR “9”).

*Example of search string (PubMed):*

***((neoplas*[TIAB] OR tumor*[TIAB] OR cancer*[TIAB] OR malignancy[TIAB]) AND***

***(2019 novel coronavirus[TIAB] OR COVID-19[TIAB] OR COVID19[TIAB] OR SARS-CoV-2[TIAB] OR 2019-nCoV[TIAB]))***

Lists of references of identified papers and reviews will be hand-searched for potentially relevant studies.

**Study review**

Data management

- Lists of studies selected at each stage included in excel files
- Abstracted variables included in excel files
- Results selected for meta-analysis included in excel files

Selection process

- Stage 1: Compile unique series of references from research
- Stage 2: Review titles, select potentially relevant papers
- Stage 3: Review abstracts of papers selected in stage 2, prepare short list for full-text review (possible original reports of studies reporting measures of severity of SarsCov2 infection on cancer patients)
- Stage 4: Full-text review of papers selected in stage 3; abstraction of data (see below)
- Stage 5: Review of lists of references of papers selected for full-text review
- All stages to be performed by four reviewers independently; results of each stage to be compared and differences to be solved by a fifth reviewer.

**Data collection**

Four reviewers will independently analyze the list of titles and abstracts, to determine if inclusion criteria are convenient and will categorize the articles with respect to the interaction between cancer and COVID-19 infection: interaction ”1” is defined as the effect that COVID-19 infection has on unvaccinated patients who  contracted the disease and concomitantly have cancer, compared to those who do not have cancer disease.

In contrast interaction “2” is defined as the effect that the pandemic due to SarsCov2 infection had on cancer: in particular, the impact it had on prevention, diagnosis and treatment of cancer disease compared to a non-pandemic period.

Finally, articles aimed at investigating a possible oncogenic effect of the infection are identified as interaction “3”. These can be in terms of associated genetic mutations or tissue alterations leading to tumors, as it is too early to assess a possible oncogenic effect of the infection itself.

Standard codes are used to report whether the cohort is within or outside the enrolled population, the type of study, and the type of cancer analyzed.

The meta-analysis will be performed according to the PRISMA-statement [Mohrer et al., 2009]. A PRISMA Flow Chart will be used to show search and selection process.

The following study characteristics for interaction ”1” will be extracted for the meta-analysis: reference, country, type of cancer, cohort size (or number of cases and controls), main results concerning SarsCov2 infection severity (relative risk and 95% confidence interval).

In case of stratified results, e.g., by age group, sex, BMI, histologic type, comorbidities, multiple entries will be included for each study.

Interaction 3 articles will be assessed in terms of the followings: impact that the infection has had at psychological level, screening and / or in-depth investigations related to a diagnosis of cancer disease, different management of treatment and follow up of the disease (postponed treatments, follow up with extended periodicity, telematic contact and not with the patient in the clinic).

In case of multiple reports from the same study, the most complete results (largest number of cases) will be used. Results for paediatric neoplasms will be excluded.

All relevant data from all eligible studies will be summarized in a standardized data extraction sheet. Data abstraction will be performed independently by four reviewers.

If the OR and/or RR are not available they will be calculated along with the respective confidence interval indicating that the data has been calculated.

**Quality assessment**

Every included article will be scored for its quality according to a standardized checklist. We will use the CASP cohort [CASP, 2018a] and the CASP case control [CASP, 2018b] checklists. The studies will be scored by answering the 12 (cohort study checklist) or 11 (case control study) answers.

**Meta-analysis**

The meta-analysis will be based on random effects models [DerSimonian & Laird, 1986], and will aim to calculate summary relative risks and 95% confidence intervals. The results of the tests for heterogeneity will be reported. In case of heterogeneity (p<0.05), its sources will be explored (e.g., histology, geographic region, study design) by conducting stratified meta-analyses.

**Evaluation of bias and confounding**

The potential effect of bias in the individual studies will be assessed by looking at the CASP score and stratifying the meta-analysis according to the score. We’ll also conduct stratified meta-analyses according to country and type of risk (reported/calculated). The presence of publication bias will be assessed with the Egger test. Particular attention will be paid to the specific sources of bias of cohort and case-control studies.

**Reporting**

The results of the meta-analysis will be reported in the peer-reviewed scientific literature.

**References**

Critical Appraisal Skills Programme (2018a). CASP Cohort Study Checklist. [online] Available at: https://casp-uk.net/casp-tools-checklists/. Accessed: 27 March 2019

Critical Appraisal Skills Programme (2018b). CASP Case Control Study Checklist. [online] Available at: https://casp-uk.net/casp-tools-checklists/. Accessed: 27 March 2019

DerSimonian R, Laird N. Meta-analysis in clinical trials. Control Clin Trials 1986;7:177-88.

Moher D, Liberati A; Tetzlaff J, Altmann DG. The PRISMA Group. Preferred Reporting Items for Systematic Reviews and Meta-Analyses: The PRISMA Statement. Ann Intern Med 2009;151:264-69.
